# Rapidly progressive glomerulonephritis revealing the coexistence of diabetic nephropathy, membranous nephropathy, and MPO-ANCA-associated crescentic glomerulonephritis 

**DOI:** 10.5414/CNCS112223

**Published:** 2026-10-13

**Authors:** Sarra Hadded, Rania Kandil, Hanene Gaied, Marwa Trabelsi, Raja Aoudia, Meriam Khadhar, Mouna Jerbi, Rim Goucha

**Affiliations:** 1 Department of Nephrology, Dialysis, and Kidney Transplantation, Mongi Slim Hospital,; 2 Faculty of Medicine, University of Tunis El Manar, and; 3 Laboratory of Renal Pathology, Charles Nicolle Hospital, Tunis, Tunisia

**Keywords:** diabetic nephropathy, membranous nephropathy, MPO-ANCA-associated glomerulonephritis, kidney biopsy, rituximab

## Abstract

Diabetic nephropathy (DN) is the leading cause of chronic kidney disease worldwide. However, Patients with diabetes presenting with atypical renal manifestations may harbor superimposed non-diabetic kidney disease, making kidney biopsy essential for accurate diagnosis. Although cases of DN coexisting with either membranous nephropathy (MN) or MPO-ANCA-associated vasculitis have been reported, the concurrent occurrence of all three entities is exceptionally rare. We report the case of a 59-year-old man with a 20-year history of poorly controlled type 2 diabetes mellitus, diabetic retinopathy, hypertension, and chronic kidney disease attributed to DN, who presented with rapidly progressive kidney dysfunction. Serum creatinine increased from a baseline of ~ 220 to 560 μmol/L, accompanied by nephrotic-range proteinuria (9 g/day) and microscopic hematuria. Immunological testing demonstrated strongly positive MPO-ANCA (120 U/mL by ELISA), whereas PR3-ANCA, anti-glomerular basement membrane, antinuclear, and anti-phospholipase A2 receptor antibodies were negative. Kidney biopsy demonstrated nodular diabetic glomerulosclerosis, membranous nephropathy, and pauci-immune crescentic glomerulonephritis consistent with MPO-ANCA-associated vasculitis. Treatment with intravenous methylprednisolone, oral prednisone, and two 1-g infusions of rituximab resulted in partial recovery of kidney function, allowing discontinuation of temporary hemodialysis and stabilization of serum creatinine at ~ 300 μmol/L. This case highlights the indispensable role of kidney biopsy in diabetic patients with rapidly progressive kidney dysfunction and illustrates the diagnostic and therapeutic implications of identifying multiple coexisting glomerular diseases.

## Introduction 

Diabetic nephropathy (DN) is the leading cause of chronic kidney disease worldwide. Nevertheless, a substantial proportion of patients with diabetes presenting with atypical renal manifestations have non-diabetic kidney disease (NDKD), either alone or superimposed on DN. Kidney biopsy studies have reported NDKD in up to 60 – 70% of selected diabetic patients undergoing renal biopsy [1]. Among these conditions, membranous nephropathy (MN) is one of the most frequently identified glomerular diseases, accounting for approximately 3 – 24% of biopsy-proven NDKD in patients with type 2 diabetes mellitus [2, 3]. 

The coexistence of DN and MN poses important diagnostic challenges because both disorders may present with nephrotic-range proteinuria. In contrast, MN associated with ANCA-associated crescentic glomerulonephritis is an uncommon clinicopathological entity. Although each of these associations has been described individually, the simultaneous occurrence of biopsy-proven DN, MN, and MPO-ANCA-associated crescentic glomerulonephritis appears to be exceptionally rare. 

We report the case of a patient with type 2 diabetes presenting with rapidly progressive glomerulonephritis and nephrotic-range proteinuria, and whose kidney biopsy demonstrated the coexistence of DN, MN, and MPO-ANCA-associated crescentic glomerulonephritis, resulting in a major change in diagnosis and management. 

## Case report 

A 59-year-old man with a 20-year history of type 2 diabetes mellitus complicated by diabetic retinopathy, hypertension, and stage 3 chronic kidney disease attributed to DN was referred to our nephrology department for rapidly progressive renal impairment. His medical history was also notable for morbid obesity, obstructive sleep apnea syndrome, and recurrent erysipelas. Diabetes control had been consistently poor, with glycated hemoglobin values ranging from 11 to 13% during follow-up. His long-term medications included insulin, lercanidipine, dapagliflozin, and atorvastatin. 

Diabetic kidney disease had been diagnosed in 2019, when the serum creatinine level was 136 μmol/L (estimated glomerular filtration rate (eGFR), ~ 50 mL/min/1.73m^2^). Renal function subsequently declined gradually and remained stable ~ 220 μmol/L (eGFR, ~ 29 mL/min/1.73m^2^) for several years. During follow-up, 24-hour urinary protein excretion remained between ~ 1.5 and 3 g/day, without hypoalbuminemia or clinical nephrotic syndrome, and was therefore considered consistent with DN. 

On admission, physical examination revealed poorly controlled hypertension and bilateral lower-extremity edema. There were no pulmonary manifestations, including dyspnea, hemoptysis, hypoxemia, or crackles on chest auscultation. Urine dipstick testing showed hematuria (2+) and proteinuria (3+). Laboratory investigations demonstrated severe renal impairment with a serum creatinine level of 560 μmol/L. The patient had normocytic normochromic non-regenerative anemia with a hemoglobin level of 9.8 g/dL, whereas leukocyte and platelet counts were within normal limits. Serum albumin was 33 g/L, and 24-hour urinary protein excretion was 9 g/day, consistent with nephrotic-range proteinuria. 

Immunological testing revealed negative antinuclear antibodies (ANA), anti-glomerular basement membrane (anti-GBM) antibodies, PR3-ANCA, and anti-phospholipase A2 receptor (PLA2R) antibodies. MPO-ANCA was strongly positive by enzyme-linked immunosorbent assay (ELISA) (120 U/mL; reference range, 0 – 10 U/mL). Serum complement levels were within the normal range (C3, 1.0 g/L; C4, 0.3 g/L). Chest computed tomography showed no evidence of pulmonary involvement or other radiological features suggestive of ANCA-associated vasculitis. Given these findings, a kidney biopsy was performed. 

The kidney biopsy specimen contained 16 glomeruli, all available for evaluation. Light microscopy showed nodular diabetic glomerulosclerosis (Kimmelstiel–Wilson lesions) with exudative lesions, including capsular drops and fibrin caps, characteristic of DN (Figure 1A). Two glomeruli exhibited segmental fibrocellular crescents superimposed on diabetic nodular glomerulosclerosis (Figure 1B). Jones methenamine silver stain demonstrated diffuse glomerular basement membrane thickening with focal spike formation, consistent with MN (Figure 1C). Moderate hyaline arteriolosclerosis was present together with interstitial fibrosis and tubular atrophy involving ~ 50% of the renal parenchyma. Immunofluorescence revealed diffuse granular capillary wall deposits of IgG and C3 (both 3+), a distribution supporting the diagnosis of MN rather than nonspecific immunoglobulin trapping occasionally encountered in DN (Figure 1D). 

The patient had no other extra-renal manifestations of vasculitis. Transthoracic echocardiography and otorhinolaryngological examination were unremarkable, and no digestive or cutaneous involvement was identified. 

A comprehensive evaluation for secondary MN was negative. The patient denied chronic exposure to non-steroidal anti-inflammatory drugs, herbal remedies, dietary supplements, over-the-counter medications, or other agents associated with secondary MN, including alpha-lipoic acid. Serological testing for hepatitis B virus, hepatitis C virus, human immunodeficiency virus, and syphilis was negative. There was no clinical or radiological evidence of malignancy. Upper gastrointestinal endoscopy and colonoscopy showed no abnormalities suggestive of gastrointestinal neoplasia. 

Induction therapy consisted of intravenous methylprednisolone (1 g daily for three consecutive days), followed by oral prednisone at 1 mg/kg/day, which was gradually tapered after 1 month. Rituximab was administered as 2 intravenous infusions of 1 g given 15 days apart. During hospitalization, the patient required 3 sessions of intermittent hemodialysis because of severe acute kidney injury, after which dialysis could be discontinued. 

Seven days after the first rituximab infusion, serum creatinine had decreased from 560 to 530 μmol/L and further declined to 503 μmol/L before the second infusion. One month after hospitalization, serum creatinine was 460 μmol/L, and at 3 months it had improved to ~ 300 μmol/L (eGFR, ~ 20 mL/min/1.73m^2^). Renal function subsequently remained stable without further need for kidney replacement therapy (Figure 2). 

## Discussion 

The coexistence of DN with another glomerular disease is a well-recognized diagnostic challenge. In diabetic patients, worsening kidney function and proteinuria are often attributed to DN, potentially delaying the diagnosis of superimposed NDKD. A pooled meta-analysis reported wide prevalence ranges for isolated DN, isolated NDKD, and mixed DN/NDKD [4]. In a large review of biopsied patients with type 2 diabetes, DN alone accounted for 41.3% of cases, isolated NDKD for 40.6%, and mixed DN/NDKD for 18.1% [1]. These findings emphasize the importance of kidney biopsy when the clinical presentation is atypical for DN. 

The association between DN and MPO-AAV is uncommon but clinically significant [5, 6]. Patients typically present with features not fully explained by DN, such as rapidly progressive renal failure, hematuria, pulmonary involvement, or elevated MPO-ANCA titers. Histologically, chronic diabetic lesions coexist with pauci-immune necrotizing and crescentic glomerulonephritis, the latter being the main driver of acute renal deterioration. Consequently, treatment is primarily directed at the vasculitic component, while supportive management of DN is maintained [7]. 

DN may also coexist with MN, which is among the most frequently reported NDKD in diabetic patients [1, 8]. Histologically, diabetic nodular glomerulosclerosis and MN may coexist within the same biopsy, making careful pathological evaluation essential. Distinguishing MN from nonspecific immunoglobulin trapping seen in DN is important because treatment is guided by the activity and risk profile of MN, whereas DN management relies on nephroprotective supportive measures [9]. 

The coexistence of MPO-AAV and MN is much rarer and has been described mainly in case reports and small cohorts [10, 11, 12]. A retrospective study identified 27 cases of ANCA-associated glomerulonephritis with MN among 223 patients with ANCA-associated glomerulonephritis [13]. Although uncommon, this overlap may be under-recognized unless electron microscopy and immunostaining are routinely performed. 

The kidney biopsy typically demonstrates two distinct pathological patterns: necrotizing crescentic glomerulonephritis with minimal immune deposits and a membranous pattern characterized by granular IgG/C3 staining and subepithelial deposits [11, 14]. Some studies have reported atypical findings, including lower PLA2R positivity, non-IgG4-dominant deposits, or glomerular MPO deposition, suggesting that some cases may represent an MPO-related immune-complex process rather than coincidental primary MN [13, 15, 16]. In our patient, immunohistochemical staining for target antigens such as PLA2R or NELL1, as well as IgG subclass analysis, was not available in our institution. Although these investigations may help characterize the underlying etiology of MN, the diagnosis itself was supported by the characteristic histopathological findings, including diffuse granular capillary wall IgG and C3 deposits and focal spike formation on Jones methenamine silver stain. 

The pathogenesis of MPO-AAV associated with MN remains uncertain. Proposed mechanisms include a coincidental association, ANCA-mediated glomerular injury promoting immune-complex formation or MPO acting as a planted antigen within the glomerular capillary wall, leading to in situ immune-complex deposition [15]. Drug-induced immune dysregulation, particularly related to levamisole-adulterated cocaine, has also been implicated [17]. The absence of circulating PLA2R antibodies in our patient supports the possibility that MN was secondary to immune dysregulation associated with MPO-AAV, although a coincidental association cannot be excluded. 

From a therapeutic perspective, MN may contribute to nephrotic-range proteinuria and persistent proteinuria despite control of vasculitic activity. However, when active crescentic glomerulonephritis is present, treatment generally follows AAV protocols [8]. In our patient, rituximab was preferred because it represents an effective induction therapy for ANCA-associated vasculitis while also being an established treatment for MN. Available data suggest that prognosis may be poorer than in isolated MN and possibly worse than in pauci-immune ANCA-associated glomerulonephritis alone [11, 14] 

Although DN may coexist with MN or ANCA-associated vasculitis, and MN has occasionally been reported in association with MPO-ANCA-associated crescentic glomerulonephritis, the simultaneous occurrence of all three biopsy-proven entities appears exceptionally rare. To the best of our knowledge, no previous case of biopsy-proven DN, MN and MPO-ANCA-associated crescentic glomerulonephritis has been reported. This case highlights the importance of kidney biopsy in diabetic patients presenting with atypical renal manifestations, particularly rapidly progressive kidney dysfunction, as it may uncover multiple coexisting glomerular diseases requiring specific treatment. 

## Conclusion 

In conclusion, rapidly progressive kidney dysfunction in patients with diabetes should not automatically be attributed to DN. Kidney biopsy remains indispensable in atypical presentations because it may reveal multiple coexisting glomerular diseases with distinct pathophysiological mechanisms and major therapeutic implications. Early recognition of these overlapping entities is essential to guide appropriate immunosuppressive therapy and improve renal outcomes. 

## Informed consent statement 

Written informed consent was obtained from the patient for the publication of this case report and the accompanying images. 

## Authors’ contributions 

Sarra Hadded: Patient management, data collection, manuscript writing, and final approval. Rania Kandil: Assistance with data collection, literature review, manuscript revision, and final approval. Hanene Gaied: Patient management, clinical follow-up, and final approval. Marwa Trabelsi: Patient management, clinical follow-up, and final approval. Raja Aoudia: Histopathological evaluation of the kidney biopsy, histological figure preparation, and final approval. Meriem Khadhar: Manuscript supervision, critical revision, and final approval. Mouna Jerbi: Manuscript supervision, critical revision, and final approval. 

## Funding 

None. 

## Conflict of interest 

The authors declare no conflict of interest. 

**Figure 1.**
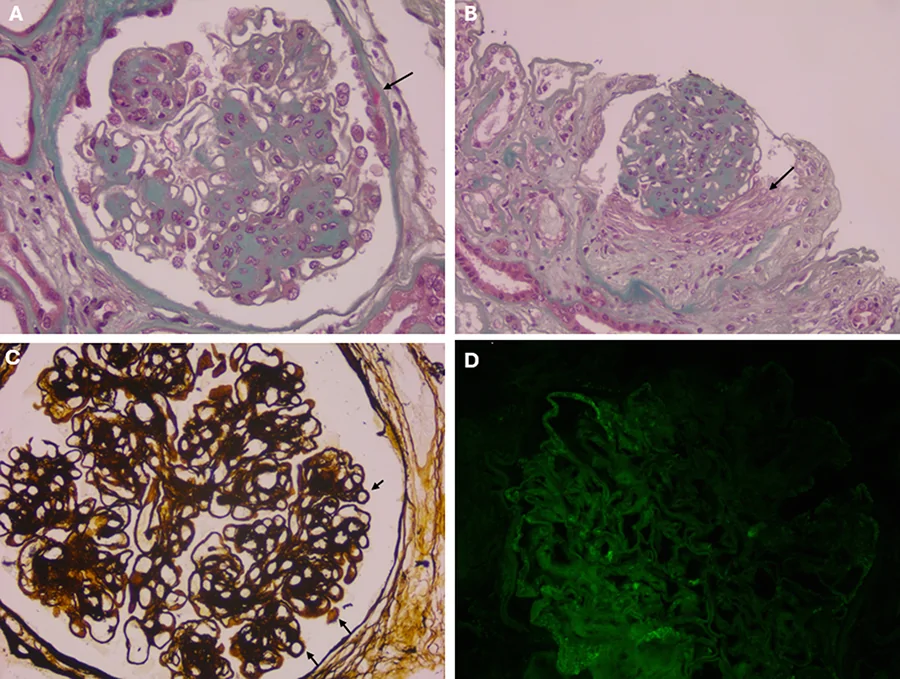
Representative kidney biopsy findings. A: Masson’s trichrome stain (× 400) showing nodular diabetic glomerulosclerosis (Kimmelstiel–Wilson lesions) with diffuse mesangial matrix expansion and a capsular drop (arrow). B: Masson’s trichrome stain (× 200) demonstrating a segmental fibrocellular crescent (arrow) superimposed on diabetic nodular glomerulosclerosis. C: Jones methenamine silver stain (× 400) showing diffuse glomerular basement membrane thickening with focal spike formation (arrows), consistent with membranous nephropathy. D: Immunofluorescence showing diffuse granular capillary wall staining for IgG (3+), consistent with membranous nephropathy.

**Figure 2.**
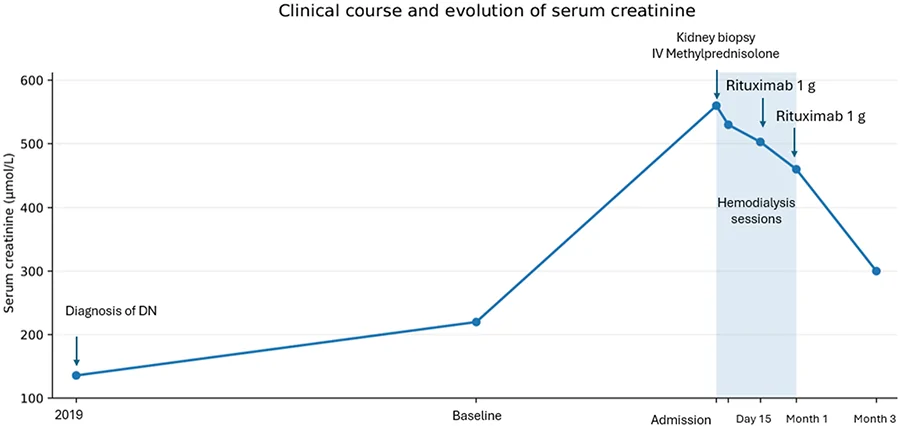
Clinical course and evolution of serum creatinine from the diagnosis of diabetic nephropathy to the last follow-up, illustrating the rapid deterioration in kidney function, kidney biopsy, initiation of immunosuppressive therapy, temporary hemodialysis, and subsequent partial renal recovery.
